# Detection of canine distemper virus (CDV) neutralising antibodies in small ruminants during peste-des-petits-ruminants virus (PPRV) surveillance in Zambia

**DOI:** 10.1186/s12917-025-04732-w

**Published:** 2025-04-30

**Authors:** Sara Lysholm, Nicola Logan, Johanna F Lindahl, Mikael Berg, Elin Johansson, Pernilla Karlsson Bergkvist, George Dautu, Ricky Chazya, Brian J. Willett, Musso Munyeme, Jonas Johansson Wensman

**Affiliations:** 1 Department of Clinical Sciences, Swedish University of Agricultural Sciences, 75007 Uppsala, Sweden; 2 Animal and Human Health Program, Department of Biosciences, International Livestock Research Institute, Nairobi, 00100 Kenya; 3 Centre for Virus Research, MRC-University of Glasgow, University of Glasgow, Glasgow, G611QH UK; 4 Department of Medical Biochemistry and Microbiology, Uppsala University, Uppsala, 75123 Sweden; 5 Department of Animal Health and Antimicrobial Strategies, Swedish Veterinary Agency, Uppsala, 75189 Sweden; 6 Department of Animal Biosciences, Swedish University of Agricultural Sciences, Uppsala, 75007 Sweden; 7 Department of Veterinary Services, Ministry of Fisheries and Livestock, Central Veterinary Research Institute, Lusaka, 50060 Zambia; 8 Department of Veterinary Services, National Livestock Epidemiology and Information Centre, Lusaka, 50060 Zambia; 9 Department of Disease Control, School of Veterinary Medicine, University of Zambia, Lusaka, 32379 Zambia; 10 Department of Microbiology, Swedish Veterinary Agency, Uppsala, 75189 Sweden

**Keywords:** Canine distemper, Canine distemper virus, Goats, Livestock markets, Morbillivirus, Peste-des-petits-ruminants, Peste-des-petits-ruminants virus, Sheep, Small ruminants, Zambia

## Abstract

Canine distemper virus (CDV) is endemic in Zambia, while peste-des-petits-ruminants virus (PPRV) has not been detected. This study screened 962 farmed goats and 237 small ruminants at informal markets for PPRV antibodies using c-ELISA. A subset (*n* = 29) was re-analysed with virus neutralisation assays (VNA) for antibodies to PPRV and CDV due to cross-reactivity concerns. c-ELISA indicated 6.44% PPRV seroprevalence on farms and 3.80% at markets. While VNA detected no PPRV antibodies, 45% (13/29) tested positive for CDV, possibly suggesting cross-reactivity. Given the study’s limitations, further research is needed to determine whether PPRV is present in Zambia and assess potential cross-reactivity.

**Supplementary Information:**

The online version contains supplementary material available at 10.1186/s12917-025-04732-w.

## Introduction, methods and result

Small ruminant husbandry is vital for poverty alleviation and food security in low- and lower-middle-income countries (LMIC) like Zambia [1], but its role is impeded by infectious diseases such as peste-des-petits-ruminants (PPR). PPR is a transboundary disease caused by peste-des-petits-ruminants virus (PPRV), classified as the species *Morbillivirus caprinae* in the *Morbillivirus* genus [2]. To date, PPRV has not been detected in Zambia, but the virus is endemic in neighbouring Tanzania [3] and the Democratic Republic of the Congo (DRC) [4–6], and has caused outbreaks in Angola [7]. Due to porous borders and uncontrolled cross-border livestock movements, the risk of PPRV introduction into Zambia is high [8, 9].

PPRV is associated with morbidity and case fatality rates of up to 80–100% in naïve populations as well as significant reductions in animal productivity in both naïve and endemic areas [10]. Recognising these severe impacts on livestock keepers’ livelihoods, the Food and Agriculture Organisation (FAO) and the World Organisation for Animal Health (WOAH) launched the PPR Global Control and Eradication Strategy (PPR GCES) in 2015, with the aim to eradicate PPRV by 2030 [11]. The availability of reliable diagnostic tests with high sensitivity and specificity are crucial for eradication. However, anecdotal concerns exist regarding risks for cross-reactivity between PPRV and canine distemper virus (CDV; species *Morbillivirus canis*) antibodies using commercially available diagnostic tests. CDV is closely related to PPRV, and several different species are susceptible, including for example dogs, hyenas and Sika deer [12, 13], with CDV-specific neutralising antibody activity also detected in sera from cattle [14]. Potential cross-reactivity between antibodies for PPRV and CDV have been documented [15], as both viruses share several cross-reactive epitopes on for example the nucleocapsid (N)-protein [16]. The risk for such cross-reactivity increases in countries where CDV is endemic, such as Zambia [17].

The aim of the present study was to screen for the presence of PPRV antibodies in goats in selected districts and in small ruminants at two informal small livestock markets in Zambia using competitive enzyme-linked immunosorbent assay (c-ELISA) and virus neutralization assay (VNA). Given the observed discrepancies between the two diagnostic tests, the study also aimed to test a subset of the serum samples for presence of CDV antibodies.

## Materials and methods

### The serological studies

#### Study area and design of the farm-based study conducted in 2019

**Fig. 1:**
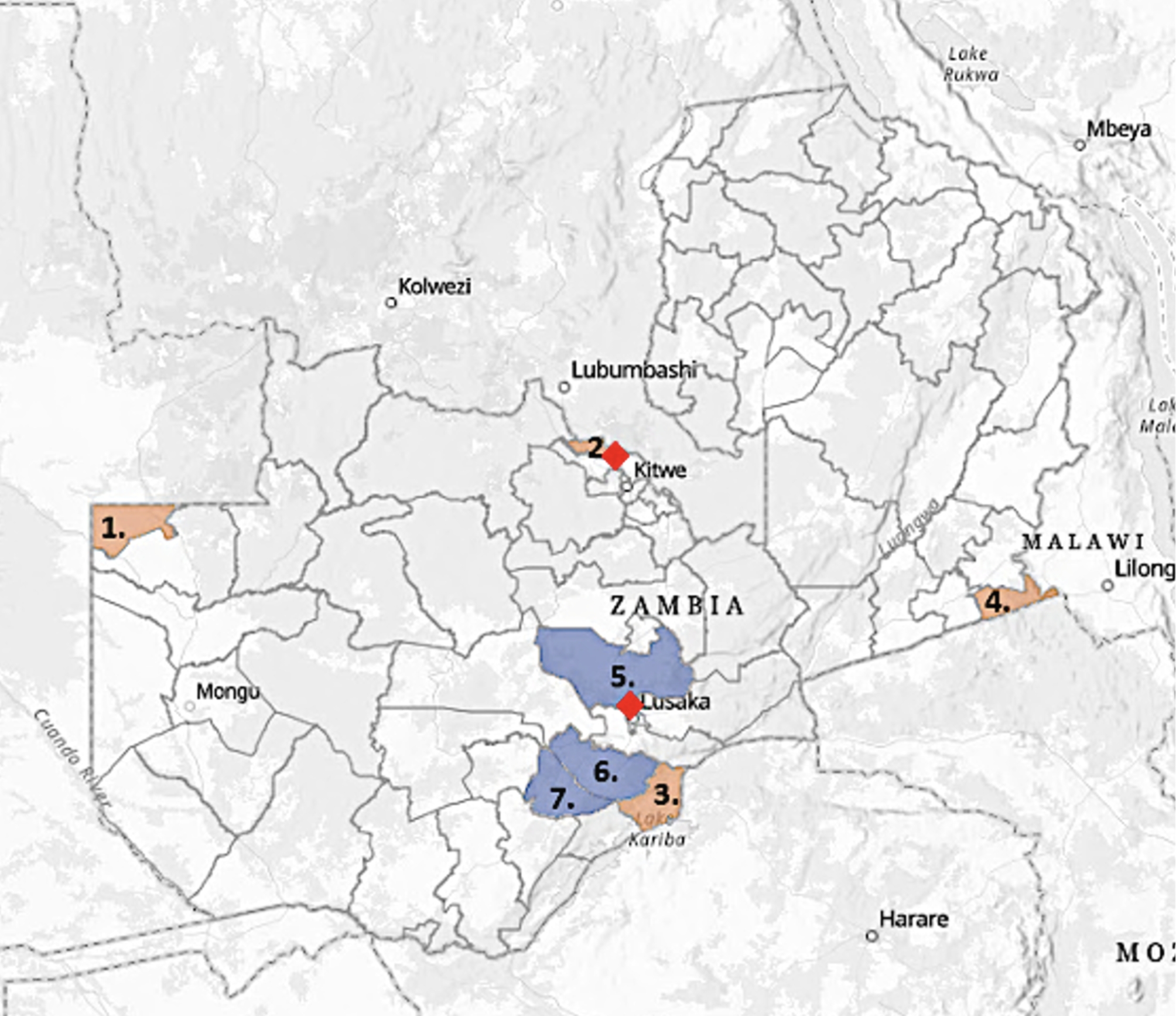
Map of Zambia with the visited districts and markets indicated. 1 = Chavuma, 2 = Chililalombwe, 3 = Siavonga, 4 = Vubwi, 5 = Chibombo, 6 = Mazabuka, 7 = Monze. The top red rhombus illustrates the location of the Kasumbalesa market, while the bottom red rhombus shows the location of the Chibolya market. Source: Esri, USGS| Esri, © OpenStreetMap contributors, HERE, Garmin, FAO, NOAA, USGS

The study was designed to provide cross-sectional data on the sero-epidemiology of selected infectious diseases in goats kept by traditional smallholder farmers in Zambia [18]. The study was conducted in September-October 2019 and included seven purposively selected districts based on location, density of goat-keeping households [19] and national and international trading activity [20, 21]. Four districts (Chavuma, Chililalombwe, Siavonga and Vubwi) were situated adjacent to one or two international borders, while the remaining three (Chibombo, Mazabuka and Monze) were located in inland Zambia (Fig. 1).

For each district, village lists were compiled by local veterinary personnel, and ten villages were selected randomly using the “randomise” tool in Microsoft Excel Version 16.58 (Microsoft, Redmond, USA). Four households per village were selected through snowball sampling [22], and 3–4 goats were sampled per household. To prevent interference with maternal antibodies, kids under four months of age were excluded from the sampling process. The study was conducted in two strata (districts with and without international borders), with the individual animal as the primary sample unit. The required sample size was determined using a simple random sampling method [23], resulting in an estimate of 444. This calculation assumed an infinite population, a 95% confidence interval, a 5% margin of error, a true prevalence of 50% and the sensitivity and specificity values of the IDVet *ID Screen PPR Competition* ELISA. The sample size was subsequently rounded upwards to 480 to allow for mistakes in sample procurement and analysis. The design effect was not estimated due to the absence of studies investigating the seroprevalence of PPRV in Zambia, and as the composition of the goat population in the country largely is unknown.

#### Study area and design of the 2018 market-based study

The study was designed to provide cross-sectional data on the seropositivity rate of selected infectious diseases in sheep and goats at the two largest small livestock markets in Zambia, namely Chibolya, located on the outskirts of a compound in Lusaka, and Kasumbalesa, situated near the border to the DRC [24] (Fig. 1). The small ruminants at the markets originated from various regions across Zambia, primarily the Southern province [20, 25], and an estimated 70–200 or more small ruminants were present at the markets at any given time [24]. Serum samples were collected at the Chibolya market over three visits over two-weeks’ time and at the Kasumbalesa market during one visit. Convenience sampling was used due to logistical challenges and the samples were collected non-randomly as the traders were in control of which animals were sampled. As the Chibolya market was visited multiple times, traders were asked to identify previously sampled animals, and careful examination of the skin around the jugular veins was conducted to prevent sampling the same animal multiple times.

#### Serum sample and data collection

In both the farm- and market-based studies, serum samples were obtained from the jugular vein and collected into serum tubes without additives (BD vacutainer, Plymouth, UK). The tubes were placed upright in a cooler box, and at the end of each day, the serum was separated and placed in a freezer at -20 °C for short term storage. Within 4–10 days the samples were transferred to -80 °C for long term storage. In the 2019 farm-based study, information on sex, age, breed, origin, and presence of clinical signs within the last twelve months and on the day of sample collection were recorded for each sampled animal. In the 2018 market-based study, information on species, sex, presence of clinical signs of disease on the day of sample collection, and details on the animal’s origin provided by the trader were recorded.

#### Competitive enzyme-linked immunosorbent assay (c-ELISA)

The sera were tested for PPRV antibodies using a commercially available competitive enzyme-linked immunosorbent assay (ELISA), namely *ID Screen PPR Competition* (ID Vet, Grabels, France), which detects antibodies to the PPRV N-protein [26]. The manufacturer reports a sensitivity and specificity of 100%; however, an independent study estimated these values to 94.5% and 99.4%, respectively, when compared to the gold standard virus neutralisation test (VNT) [26]. The kit’s positive control as well as PPR reference serum was used as positive controls, while nuclease-free water served as a negative control. The kit was used, validated and interpreted following the manufacturer’s instructions. Results were categorized as positive, doubtful or negative, and doubtful outcomes were considered negative.

#### Virus neutralization assay (VNA) for detection of antibodies to PPRV

In total, 29 of the serum samples underwent confirmatory analysis using a virus neutralisation assay (VNA) [14]. These samples were selected based on their competition percentage result from the c-ELISA analysis, covering a spectrum from strong positives to negatives, and also considering sample location to ensure representation from all seven districts and both livestock markets. In brief, replication-defective recombinant vesicular stomatitis virus (VSV) [27] that expressed the haemagglutinin (H) and fusion (F) proteins of PPRV Nigeria 75/1 [15], bearing a luciferase marker gene “VSVΔG*luc* (PPRV)”, were manufactured, and used to measure virus neutralising activity in a plate-based micro-neutralisation assay. The sera were diluted 1:50 and mixed with a fixed input of VSVΔG*luc* (PPRV) pseudotype and incubated for 1 h at 37℃, after which the sera/pseudotype mixtures were plated onto 293-DogSLAM cells and incubated for 48 to 72 h, at which time luciferase substrate was added (Steadylite plus™, Perkin Elmer, Waltham, Massachusetts, USA) and the signal analysed on an Ensight Multi-mode plate reader (Perkin Elmer, Waltham, Massachusetts, USA).

#### Virus neutralization assay (VNA) for detection of antibodies to CDV

The same subset of samples (*n* = 29) was also analysed for presence of antibodies to CDV using VSV pseudotypes bearing the H and F proteins of the Onderstepoort strain of CDV [15]. These “VSVΔG*luc* (CDV)” pseudotypes were used to measure virus neutralising activity as above. Human serum was used as negative control and serum from a dog vaccinated for CDV served as positive control. Both the positive and negative control yielded the expected results in the analysis.

## Results

### Presence of PPRV antibodies in the 2019 farm-based study using c-ELISA

Serum samples were collected from 962 goats from 280 different herds. Apparent animal-level seroprevalence was 6.44% (95% CI 4.98–8.19) with seropositive animals detected across all visited districts (Table 1). Apparent herd-level seroprevalence was 15.0% (11.0-19.7).

**Table 1:** Apparent animal-level seroprevalence for PPRV

		Positive (analysed)	Seroprevalence % (95% CI)
Total		62 (962)	6.44 (4.98–8.19)
Sex	Female	56 (806)	6.95 (5.29–8.93)
	Male	6 (151)	3.97 (1.15–8.45)
Age group	< 1 year	5 (142)	3.52 (1.15–8.03)
	1–3 years	39 (651)	5.99 (4.29–8.10)
	> 3 years	17 (158)	10.8 (6.39–16.7)
District	Chavuma	3 (122)	2.46 (0.51–7.02)
	Chibombo	40 (160)	25.0 (18.5–32.4)
	Chililalombwe	1 (122)	0.82 (0.02–4.48)
	Mazabuka	7 (160)	4.38 (1.78–8.81)
	Monze	4 (158)	2.53 (0.69–6.35)
	Siavonga	3 (120)	2.50 (0.52–7.13)
	Vubwi	4 (120)	3.33 (0.92–8.31)

### 2018 market-based serological study

#### PPRV c-ELISA seropositivity rate at the Chibolya and Kasumbalesa markets

In total, 237 serum samples were collected from goats and sheep present at the markets. Approximately 60% of the samples were obtained from the Chibolya market, with the remaining 40% from the Kasumbalesa market. Eight goats and one sheep were seropositive for PPRV, resulting in a seropositivity rate of 3.80% (95% CI 1.75–7.09) (Table 2).

**Table 2:** Seropositivity rate of PPRV antibodies at the Lusaka and Kasumbalesa markets

Variable		Positive (analysed)	% seropositive (95% CI)	
	Total	9 (237)	3.80 (1.75–7.09)	
Market	Kasumbalesa	5 (94)	5.32 (1.75-12.0)	
	Lusaka	4 (143)	2.80 (0.77–7.01)	
Species	Goats	8 (223)	3.59 (1.56–6.95)	
	Sheep	1 (14)	7.14 (0.18–33.9)	
Sex^b^	Female	6 (125)	4.80 (1.78–10.2)	
	Male	3 (108)	2.78 (0.58–7.90)	
Province^c^	Southern	6 (193)	3.11 (1.15–6.64)	
	Eastern	2 (15)	13.3 (1.66–40.5)	
	Central	0 (15)	0 (0-21.8)^a^	
	Lusaka	0 (1)	0 (0-97.5)^a^	

^a^ One-sided 97.5% confidence interval

^b^ Missing information for 4 animals

^c^ Missing information for 13 animals

### Absence of PPRV specific antibodies analysed with VNA

In 2022, 29 of the serum samples underwent re-analysis with VNA. Of these, 26 samples were positive when analysed with c-ELISA, one was doubtful and two were negative. While weak unspecific activity was initially seen in some samples, subsequent titration confirmed that none were positive (Table S1).

### Serum samples positive for CDV antibodies with adapted VNA

The same subset of samples underwent analysis for the presence of CDV antibodies using VNA. A total of 13 samples (45%) tested positive and the neutralisation percentage varied from 94.2 to 99.9%. Among the 26 samples that tested positive for PPRV antibodies with c-ELISA, 11 (42%) were positive for CDV antibodies. Both samples that had tested negative with the c-ELISA were positive for CDV antibodies, while the “doubtful” sample was negative (Table S1).

## Discussion

This study investigated the presence of antibodies to PPRV and CDV in small ruminants in Zambia. While PPRV antibodies were detected with c-ELISA, a subset of the samples tested negative for PPRV-antibodies with VNA, while some exhibited seropositivity for CDV. This marks the first report of CDV antibodies in sera from small ruminants identified as PPRV-seropositive using a commercial diagnostic test.

Although PPRV-seropositive small ruminants have been previously identified in Zambia [28, 29], there is no current evidence of active virus circulation, and it has been hypothesised that earlier seroconversions may be attributable to the introduction of vaccinated or naturally infected animals from neighbouring countries. The potential introduction of PPRV into Zambia raises concerns for adverse impacts on small ruminant health, stakeholder livelihoods and national and international trade of small ruminants and their products. Such an incursion would also represent a major setback for the global PPR eradication programme led by the FAO and WOAH. These implications underscore the importance of addressing potential false positives, including those resulting from antibody cross-reactivity between different pathogens such as PPRV and CDV. This consideration is especially important when designing control and eradication strategies in non-endemic PPRV regions, and in countries with a high dog population and endemic CDV presence, such as Zambia [17].

Canine distemper virus exhibits a broad host range extending far beyond the *Canidae* family [12, 13, 31]. Cross-neutralising activity against CDV has been observed in sera from PPRV-infected goats [14]. Studies have identified cross-reactive epitopes between PPRV and CDV, such as on the PPRV N-protein [16], which is targeted by the c-ELISA utilised in this study. While the utilised c-ELISA has high sensitivity and specificity values when compared to the gold-standard virus neutralisation test (94.5% and 99.4%, respectively) [26], anecdotal suspicions of cross-reactivity between PPRV and CDV antibodies with the c-ELISA exists. While the current study does not provide evidence of such cross-reactivity, potential indications were observed as 11 of the 26 samples that had tested positive for PPRV antibodies with c-ELISA also were positive for CDV with VNA. However, two samples that tested negative for PPRV antibodies using c-ELISA were also positive for CDV with VNA, highlighting the need for additional research investigating the risk of cross-reactivity between antibodies for PPRV and CDV in commonly used diagnostic tests. Such information would provide important insights for accurately informing future diagnostic and surveillance initiatives.

In the current study, analysis using c-ELISA detected PPRV seropositive small ruminants across all surveyed districts and age-groups, as well as at both markets. None of the farmers reported vaccinating their small ruminants, and local veterinary and para-veterinary officers confirmed the absence of PPRV vaccination campaigns in the visited areas. However, upon re-analysis with VNA, none of the 29 samples yielded positive results. The VNA is considered the gold standard diagnostic method for detecting PPRV antibodies [32] and a recent study [33] reported good agreement between c-ELISA and VNA. It should be noted that the c-ELISA used here detects antibodies for the virus nucleocapsid (N) protein [26] while the VNA targets antibodies for the hemagglutinin (H) and fusion (F) proteins [14]. The discrepancy between the two methods may therefore be due to differences in the presence, quality, and/or quantity of specific antibodies in the analysed samples. Ideally, the samples should have been tested simultaneously using an ELISA that detects antibodies against the H-protein, such as AU-PANVACs monoclonal antibody-based blocking ELISA [34], alongside the ID-Vet c-ELISA. However, this was not done in the present study.

In the current study, the VNA was conducted three years after the c-ELISA due to logistical issues stemming from the COVID-19 pandemic. During this period, the freezer in which the samples were stored broke down at least once, and frequent power outages resulted in samples thawing to varying degrees. Antibodies for certain pathogens have demonstrated resilience to prolonged storage and to repeated freeze-thaw cycles [35–37]. Long-term storage and repeated freeze-thaw cycles should however be avoided, as this may lead to antibody denaturation [37, 38]. Additionally, the samples had to be shipped from Zambia to Scotland for the VNA, requiring a heat-inactivation process prior to transport, where the samples were kept at 56 °C for two hours. The combination of long-term storage, repeated freeze-thaw cycles and heat inactivation may have affected the antibodies present in the samples. While previous research indicate maintained stability at 56 °C for IgG antibodies (targeted by the diagnostic assays included in this study) [30], retesting the samples with c-ELISA after the heat-inactivation procedure, prior or in short concession to VNA, would have provided valuable diagnostic insights. It is recommended that future studies of a similar design include this step to better assess the potential impact of heat inactivation (and long-term storage) on antibody detection.

The authors acknowledge that, ideally, PPRV VNA analysis should have been conducted in close succession to the c-ELISA analysis and on all serum samples, which unfortunately was not possible in the current study. Despite this limitation, the findings underscore the necessity for further research, not only to ascertain whether PPRV is circulating in Zambia, but also to elucidate the patterns of potential cross-reactivity between PPRV and CDV, as this is pivotal for accurate interpretation of PPRV seroprevalence data. Future PPR sero-surveillance in Zambia should prioritise high-risk areas, including border regions near infected countries and key livestock trade and movement routes. Given the high seroprevalence observed in Chibombo district with c-ELISA, future studies should consider including this district. To reduce the risk of false positive results, analysis with N-protein ELISA should be followed by a confirmatory method, ideally VNA. Where possible, serological surveillance should be complemented with direct diagnostic testing of animals exhibiting clinical signs of suggestive PPRV infection, such as RT-PCR (quantitative or conventional), antigen-ELISA or Penside tests [32]. Additionally, experimental studies should further investigate the extent of cross-reactivity between PPRV and CDV antibodies in commonly used diagnostic tests. By addressing these knowledge gaps, future research can enhance our understanding of the epidemiology and diagnostic challenges associated with morbillivirus infections in small ruminants and other susceptible species.

## Conclusion

This study reports the presence of antibodies to CDV in goats and could possibly suggest cross-reactivity with CDV antibodies in samples that tested positive for PPRV using a commercially available c-ELISA detecting antibodies targeting the N-protein. Given the study’s limitations and the ongoing global efforts to eradicate PPRV, further research is needed to comprehensively investigate the extent and implications of such potential cross-reactivity. While small ruminants of various ages and geographical origins tested positive for PPRV antibodies with c-ELISA, all 29 samples analysed with VNA were negative. These findings underscore the necessity for further research on PPRV in Zambia, ideally integrating extensive sero-surveillance studies with efforts to detect either viral antigen or genetic material.

## Electronic supplementary material

Below is the link to the electronic supplementary material.

Supplementary Material 1

## Data Availability

The datasets generated in the current study are available from the corresponding author on reasonable request.
