# Supplementary material for: Detection of canine distemper virus (CDV) neutralising antibodies in small ruminants during peste-des-petits-ruminants virus (PPRV) surveillance in Zambia
Source: BMC Vet Res. 2025 Apr 30;21:303. doi: 10.1186/s12917-025-04732-w (PMC12042581; doi:10.1186/s12917-025-04732-w)
Supplement: Supplementary file 1 — Supplementary Material 1 [file 12917_2025_4732_MOESM1_ESM.docx]

**Table S1**: Description of c-ELISA competition percentage (%) and results, and CDV VNA neutralisation (%) and results for 29 samples that were subjected to both analyses

| **ID** | **c-ELISA competition percentage (%)** | **ELISA result** | **Neutralisation (%)** | **CDV VNA result** |
| --- | --- | --- | --- | --- |
| 1 | 23.5 | Positive | 96.5 | Positive |
| 2 | 23.6 | Positive | 42.3 | Negative |
| 3 | 25.4 | Positive | 95.5 | Positive |
| 4 | 26.2 | Positive | 65.4 | Negative |
| 5 | 28.5 | Positive | 99.9 | Positive |
| 6 | 30.1 | Positive | 94.4 | Positive |
| 7 | 31.1 | Positive | 99.3 | Positive |
| 8 | 32.2 | Positive | 42.6 | Negative |
| 9 | 32.2 | Positive | 94.7 | Positive |
| 10 | 32.5 | Positive | 69.3 | Negative |
| 11 | 35.3 | Positive | 99.4 | Positive |
| 12 | 35.6 | Positive | 65.4 | Negative |
| 13 | 36 | Positive | 98.3 | Positive |
| 14 | 37.1 | Positive | 60.1 | Negative |
| 15 | 38.7 | Positive | 82.9 | Negative |
| 16 | 41.1 | Positive | 96.4 | Positive |
| 17 | 42.4 | Positive | 56.2 | Negative |
| 18 | 43.2 | Positive | 64.2 | Negative |
| 19 | 44.9 | Positive | 61.1 | Negative |
| 20 | 45.1 | Positive | 98.1 | Positive |
| 21 | 45.6 | Positive | 17.5 | Negative |
| 22 | 45.8 | Positive | 84.3 | Negative |
| 23 | 46.6 | Positive | 97.6 | Positive |
| 24 | 47.3 | Positive | 65.5 | Negative |
| 25 | 48 | Positive | 45.9 | Negative |
| 26 | 48.7 | Positive | 27.9 | Negative |
| 27 | 55.1 | Doubtful | 62.3 | Negative |
| 28 | 75.6 | Negative | 99.8 | Positive |
| 29 | 81.7 | Negative | 99.9 | Positive |
